# Zeolite-derived hybrid materials with adjustable organic pillars†
†Electronic supplementary information (ESI) available. See DOI: 10.1039/c5sc04602e


**DOI:** 10.1039/c5sc04602e

**Published:** 2016-02-09

**Authors:** Maksym Opanasenko, Mariya Shamzhy, Fengjiao Yu, Wuzong Zhou, Russell E. Morris, Jiří Čejka

**Affiliations:** a J. Heyrovský Institute of Physical Chemistry , Academy of Sciences of the Czech Republic , v.v.i., Dolejškova 3 , CZ-182 23 Prague 8 , Czech Republic . Email: jiri.cejka@jh-inst.cas.cz; b EaStChem School of Chemistry , The University of St Andrews , St Andrews , KY16 9ST , Scotland , UK . Email: rem1@st-andrews.ac.uk

## Abstract

Porous organic–inorganic materials with tunable textural characteristics were synthesized using the top-down process by intercalating silsesquioxanes and polyhedral oligomeric siloxanes of different types between crystalline zeolite-derived layers. The influence of key parameters such as (i) linker nature (pure hydrocarbon, S-, N-containing); (ii) chain length in alkyl- and aryl bis(trialkoxysilyl) derivatives; (iii) denticity of the organic precursor molecules; (iv) nature and size of side chain in mono(trialkoxysilyl) substrates; (v) rigidity of the chain (saturated *vs.* unsaturated, aliphatic *vs.* aromatic); (vi) nature and size of leaving group on the structural and textural properties of formed hybrids was carefully addressed. It was established, that the optimal silsesquioxane appropriate for the formation of zeolite-derived hybrids with high textural characteristics should possess short alkyl or long aryl chains, relatively small leaving groups and denticity larger than 3. Addition of polydentate low-molecular binder improved the structural and textural characteristics of hybrids, especially when using bulky or hydrophilic linkers.

## Abstract 2

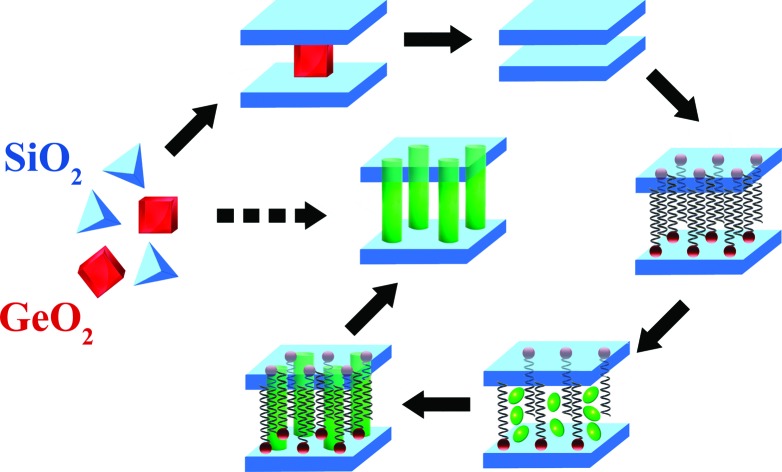
Porous organic–inorganic materials with tunable textural characteristics were synthesized using the top-down process by intercalating silsesquioxanes and polyhedral oligomeric siloxanes of different types between crystalline zeolite-derived layers.

## 1. Introduction

Porous hybrid nanostructures and composites are a class of materials, which may not only combine the advantages of their individual components, but also possess new properties useful for practical applications. During the last decade, two main groups of hybrid materials bearing organic and inorganic constituents have been the subject of deep investigation in the field of materials science: metal–organic frameworks^1^,^2^ and periodic organosilicas (both micro- and mesoporous).^3^,^4^ Besides the post-synthetic grafting known as a method for design of hybrid materials,^5^ most of the reported techniques for the preparation of organic–inorganic nanostructures and composites are based on direct synthesis.^6^,^7^ In general, such an approach assumes the self-organisation of the respective building-blocks (most frequently, in the presence of structure-directing agent – SDA) to form predetermined structures with predestined characteristics and does not allow detailed control over the structural properties or porosity of final material. In particular, a set of hybrid ECS materials consisting of zeolite-like inorganic layers and bridged organic disilanes have been obtained by direct synthesis during the last decade.^8^–^10^ Such ECS materials can be considered as crystalline layers pillared by covalent bonding with respective organic groups (*e.g.* –C_6_H_4_–, –C_6_H_4_–C_6_H_4_–, –(CH_2_)_2_–C_6_H_4_–(CH_2_)_2_– or –(CH_2_)_3_– *etc.*).

**Fig. 1:**
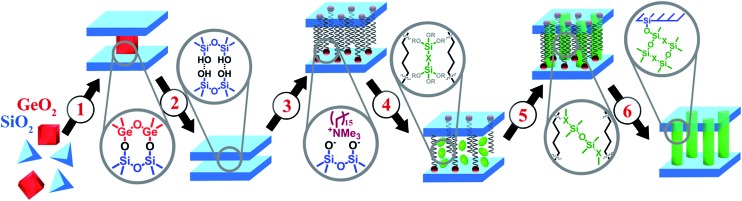
Illustration of the approach based on using of zeolite precursor and organic building-blocks. Intermediate stages: (1) synthesis; (2) disassembly; (3) swelling; (4) intercalation of organosilicas; (5) hydrolysis of precursor; (6) removal of swelling agent.

A recently developed method for the top-down synthesis of zeolitic, 2D silica layers through disassembly of 3D zeolites has enlarged the set of possible inorganic building units appropriate for the design of novel organic–inorganic materials. In this method, inert 2D silica layers (IPC-1P) are formed through removal of layered building units, *e.g.* Ge-enriched double four ring units, D4Rs, present in the parent UTL zeolite.^11^–^13^ The use of UTL-derived IPC-1P layered material has allowed the production of organic–inorganic hybrids with tunable textural properties.^14^ A similar approach was used for the preparation of hybrid materials from directly obtained layers with MWW^15^ and MFI^16^ topology. The main idea of manipulation with the layers and interlayer connections is illustrated on Fig. 1. We start with the synthesis of the zeolite structure, which may be disassembled into discrete parts (1 on Fig. 1). Successful disassembly of the framework of appropriate zeolite provides uniform fragments (2 on Fig. 1), which can be reassembled directly or in the combination with other “building-blocks” to form new structures. A swelling step (3 on Fig. 1) is required for the efficient separation of the layers allowing further intercalation of the inorganic/organic component (4 on Fig. 1) followed by precursor hydrolysis (5 on Fig. 1) and swelling agent extraction (6 on Fig. 1) steps. Hybrid materials containing functional groups were obtained using such approach and successfully applied in catalysis.^17^ The distinctive feature of organic–inorganic materials prepared using the described approach is the possibility to vary their textural properties (*i.e.* surface area, volume of micro- and mesopores) by changing synthesis parameters such as the ratio of the reagents or type of the linker.^14^

Taking into account the potential of hybrid materials for applications in separation^18^,^19^ and catalysis,^3^,^20^–^22^ and the advantages of top-down method for fabrication of novel porous organic–inorganic materials, in this contribution we focus on the detailed investigation of the relation between the nature of organic building-blocks (*e.g.* type, size, denticity, rigidity, *etc.*) and physico-chemical properties (structure, porosity) of formed hybrid zeolite-based materials. A variety of organic ligands was used for preparation of the target hybrids and a comparative analysis of their characteristics (Table 1).

**Table 1:** List of precursors used for the synthesis of hybrids

Full name	Structural formula	Abbreviation
Bis(triethoxysilyl)methane	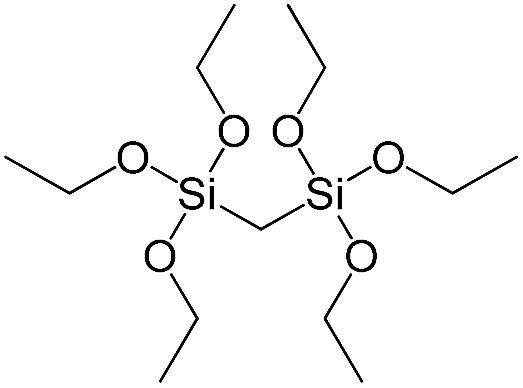	S1
1,2-Bis(triethoxysilyl)ethane	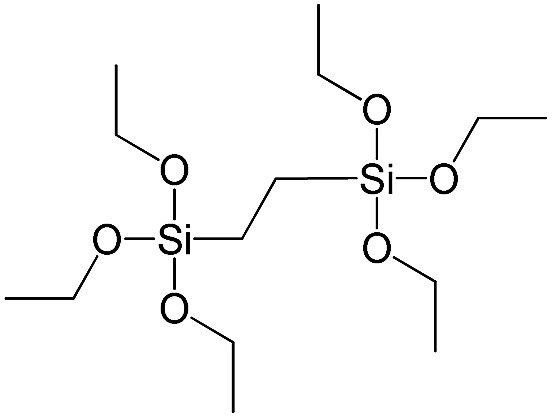	S2
1,2-Bis(trimethoxysilyl)ethane	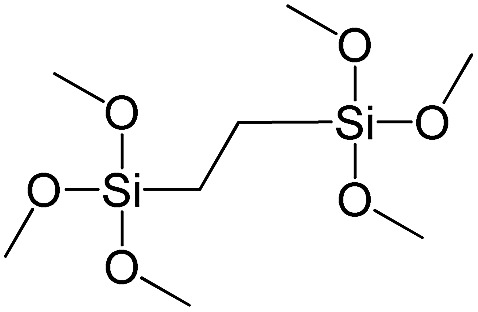	S3
1,8-Bis(triethoxysilyl)octane	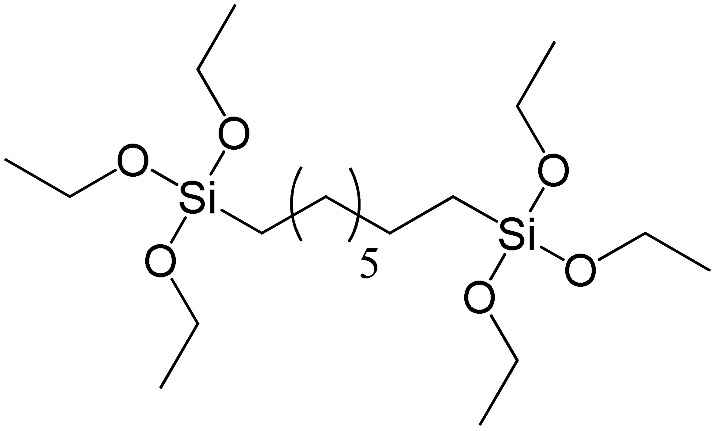	S4
1,4-Bis(triethoxysilyl)benzene	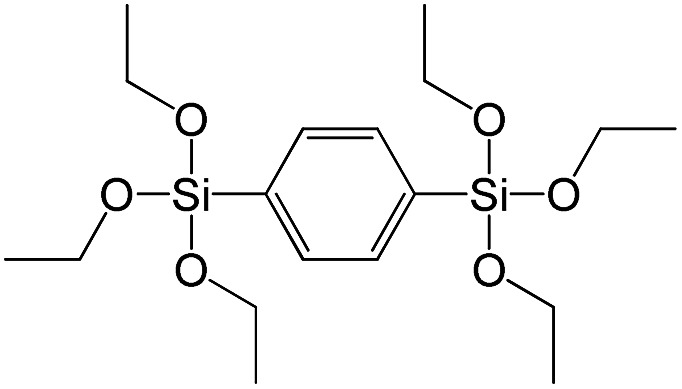	S5
1,3-Bis(triethoxysilyl)benzene	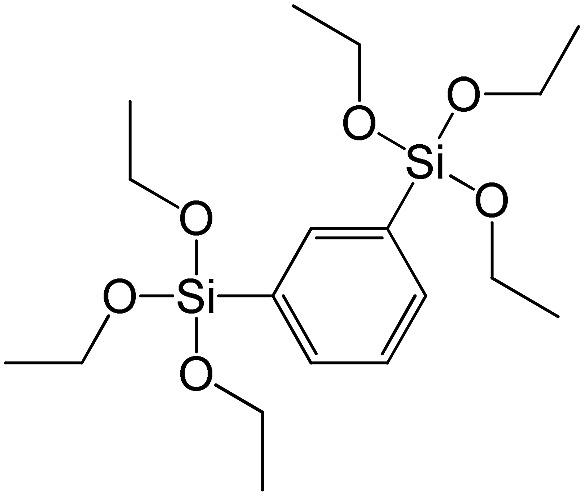	S6
4,4-Bis(triethoxysilyl)-1,1′-biphenyl	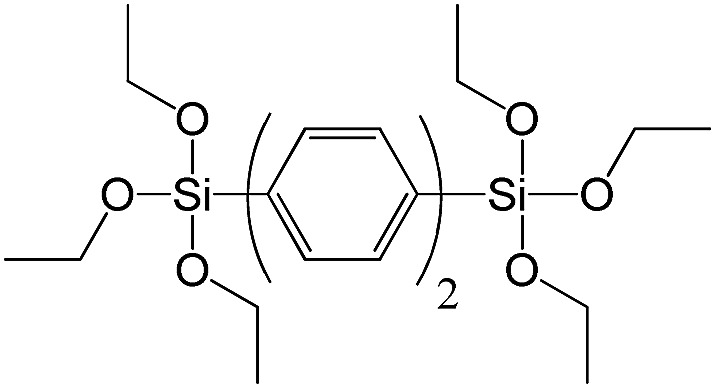	S7
1,2-Bis(triethoxysilyl)ethene	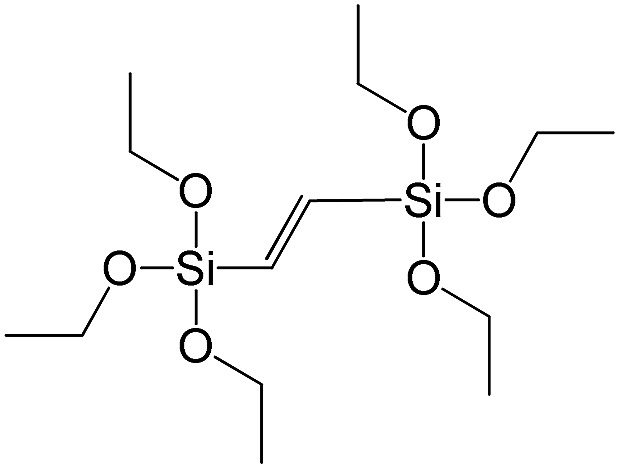	S8
Bis[3-(triethoxysilyl)propyl]tetrasulfide	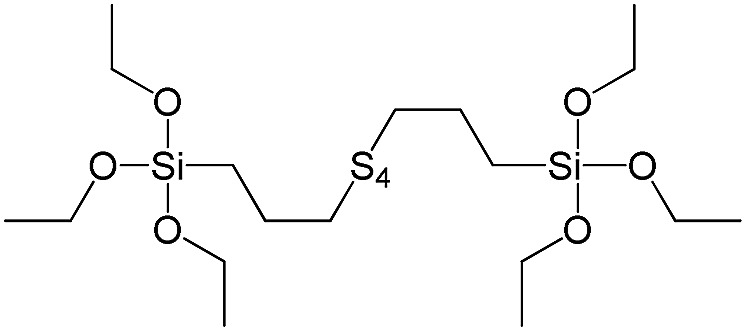	S9
Bis[3-(trimethoxysilyl)propyl]-*N*-methylamine	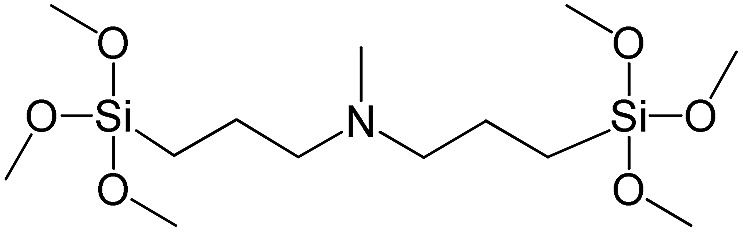	S10
*N*-[3(Trimethoxysilyl)propyl]ethylenediamine	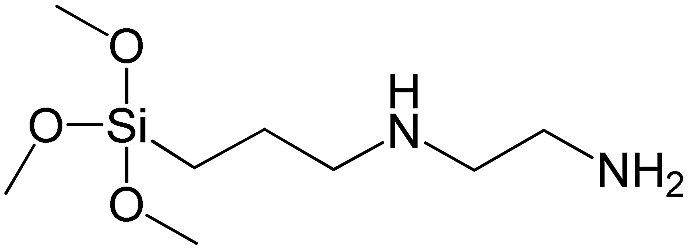	S11
Vinyltrimethoxysilane	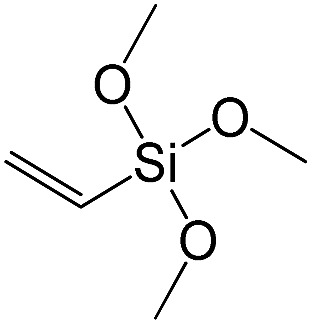	S12
Triethoxyphenylsilane	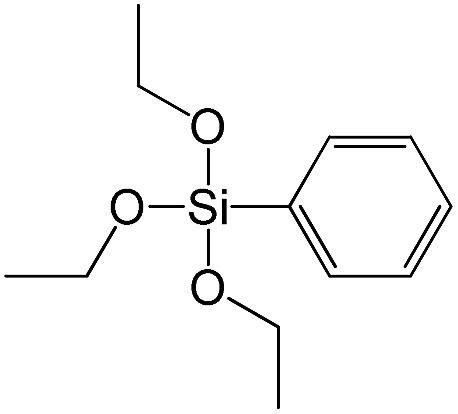	S13
Triethoxy-*p*-tolylsilane	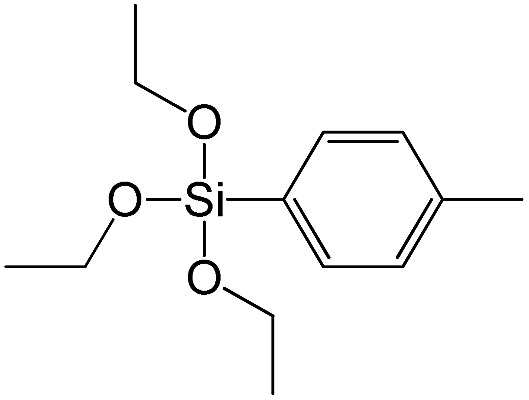	S14
(4-Biphenyl)triethoxysilane	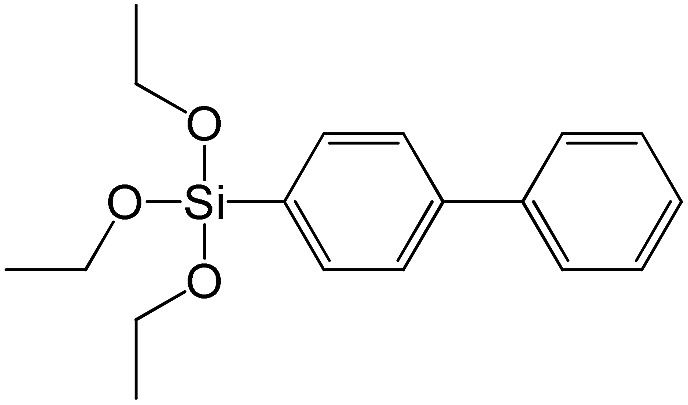	S15
(Pentafluorophenyl)triethoxy-silane	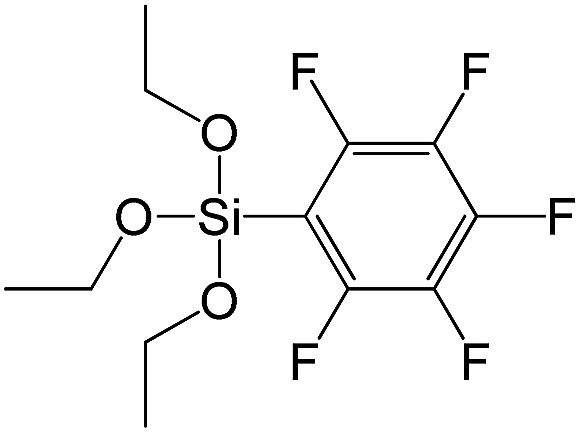	S16
Dimethoxydiphenylsilane	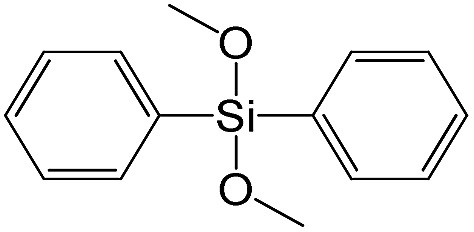	S17
PSS-hydrate-octakis(tetramethylammonium) substituted	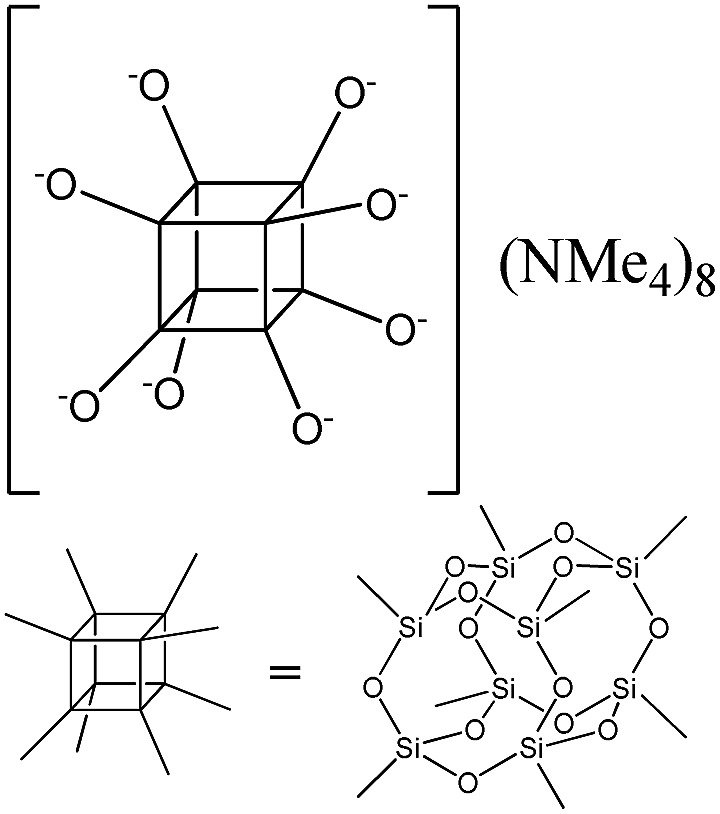	P1
PSS-octa(2-trichlorosilylethyl) substituted	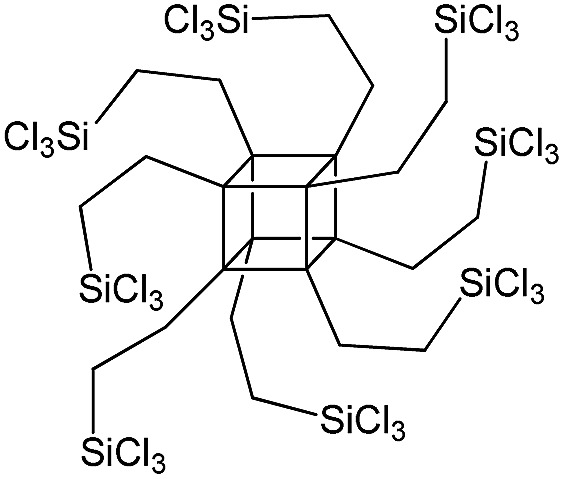	P2
PSS-octakis(dimethylsilyloxy) substituted	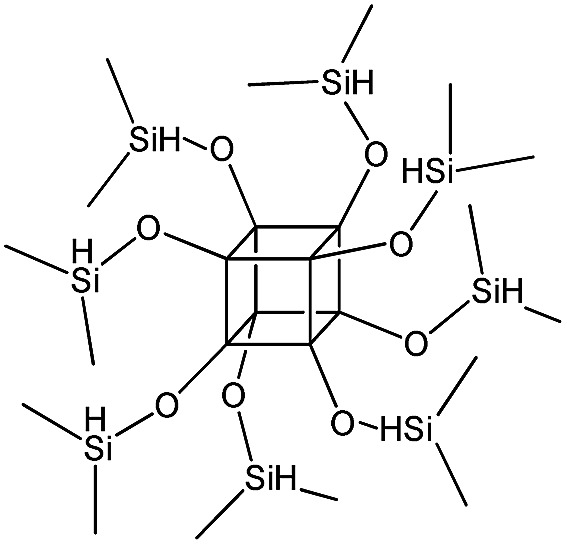	P3
PSS-octakis[2-(chlorodimethylsilyl)ethyl] substituted	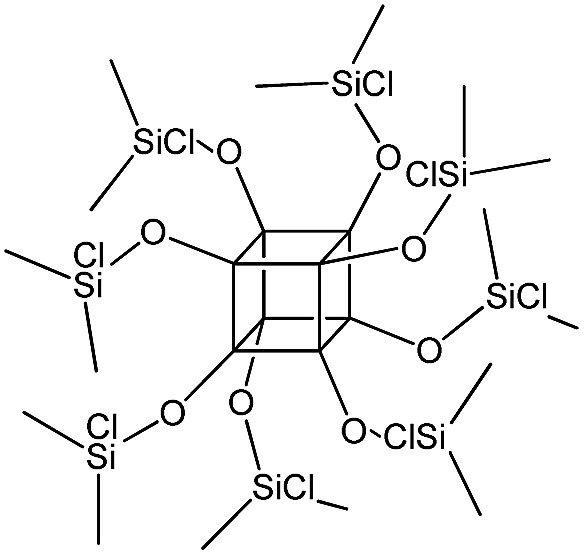	P4
PSS-octamethyl substituted	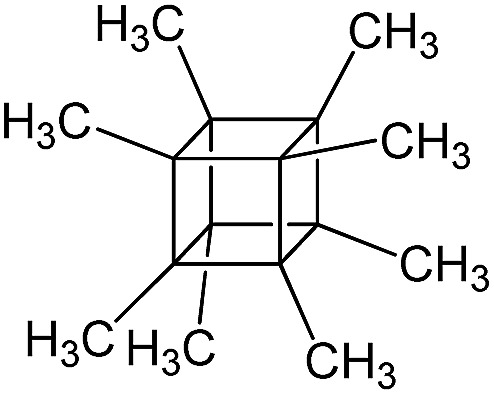	P5
PSS-octaphenyl substituted	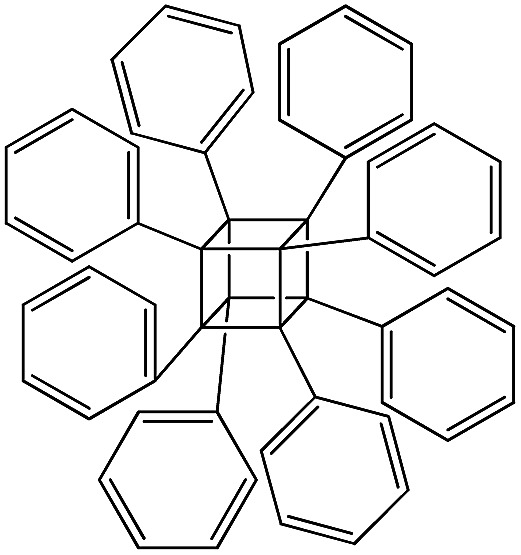	P6

## 2. Experimental

### 2.1. Synthesis of layered precursor IPC-1P

Preparation of 7-ethyl-6-azoniaspiro-[5.5]-undecane hydroxide (SDA for the synthesis of zeolite UTL) and following synthesis of UTL zeolite were carried out using methods similar to ref. 23 IPC-1P precursor was prepared as described in ref. 14 calcined UTL was hydrolysed in 0.1 M HCl with the w/w ratio 1/200 at 90 °C overnight. The product was isolated by centrifugation, washed with distilled water, and dried at 60 °C.

### 2.2. Swelling of IPC-1P precursor

The swollen material, IPC-1SW, was prepared by treating IPC-1P with a mixture of 40 wt% tetrapropylammonium hydroxide (TPA-OH) and 25 wt% hexadecyltrimethylammonium chloride (CTMA-Cl) (w/w = 1/9) in the ratio 1/50 (w/w). The mixture was stirred at ambient temperature overnight (10 h). The product was isolated by centrifugation, washed out with water, and dried at 80 °C.

### 2.3. Synthesis of pillared materials using silsesquioxanes (Sn) and polyhedral oligomeric siloxanes (Pn)

IPC-1SW (0.2 g) was vigorously stirred with a chloroform solution (5 ml) of 0.2–0.4 g of respective reagent for 2 days at 60 °C. Solvent was partially evaporated at 40 °C and 20 torr. Mixture was treated with water for 2 days at 80 °C. The white solid separated by centrifugation was dried for 1 day at 65 °C.

### 2.4. Removal of the swelling agent

To remove the CTMA, the pillared material (0.2 g) was suspended in 30 ml of 1 M NH_4_NO_3_ solution in ethanol/H_2_O (w/w = 1/1) for 2 days at room temperature (ammonia salt was used for ionic exchange with CTMA cations, ethanol was used for the shift of respective equilibrium due to the increasing of CTMA solubility). The solid, separated by centrifugation, was treated with 0.2 M HCl solution in ethanol/octane mixture (w/w = 1/1) for 2 days at 60 °C. The final product was filtered off, washed with water, ethanol/octane (w/w = 1/1) solution, ethanol and then dried at 65 °C overnight.

### 2.5. Characterization

X-ray powder diffraction data were collected on a Bruker AXS D8 Advance diffractometer with a Vantec-1 detector in the Bragg–Brentano geometry using CuKα (*λ* = 1.54178 Å) radiation.

Adsorption isotherms of nitrogen at –196 °C were recorded using an ASAP 2020 (Micromeritics) static volumetric apparatus. Before adsorption, the samples were degassed with a turbomolecular pump at 200 °C for 8 h.

The microstructures were investigated using high resolution transmission electron microscopy (HRTEM) on a Jeol JEM-2011 electron microscope operating at an accelerating voltage of 200 kV. The HRTEM images were recorded using a Gatan 794 CCD camera. The camera length, sample position and magnification were calibrated using standard gold film methods.

The Knoevenagel condensation of benzaldehyde and malononitrile was performed in liquid phase under atmospheric pressure and temperature of 25 °C in a multi-experiment work station StarFish (Radley's Discovery Technologies UK). The reaction products were analyzed by gas chromatography (GC) using an Agilent 6850 with FID detector equipped with a nonpolar HP1 column (diameter 0.25 mm, thickness 0.2 μm and length 30 m). Reaction products were indentified using GC-MS analysis (ThermoFinnigan, FOCUS DSQ II Single Quadrupole GC/MS).

## 3. Results and discussion

To understand the influence of the type and nature of organic precursors on the properties of hybrid materials, silsesquioxanes (S1–S17, Table 1) and polyhedral oligomeric siloxanes (P1–P6, Table 1) have been grouped regarding:

• linker nature (pure hydrocarbon, S-, N-containing);

• chain length in alkyl- and aryl bis(trialkoxysilyl) derivatives;

• denticity of the organic precursor molecules (from 2 to 6);

• nature and size of side chain in mono(trialkoxysilyl) substrates;

• rigidity of the chain (saturated *vs.* unsaturated, aliphatic *vs.* aromatic); nature and size of condensing (leaving) group.

In addition, we addressed the influence of the ratio between inorganic and organic components, and presence of low-molecular binding additives (*e.g.* TEOS) on structural and textural properties of UTL-derived hybrids.

### 3.1. Linker nature and chain length

**Fig. 2:**
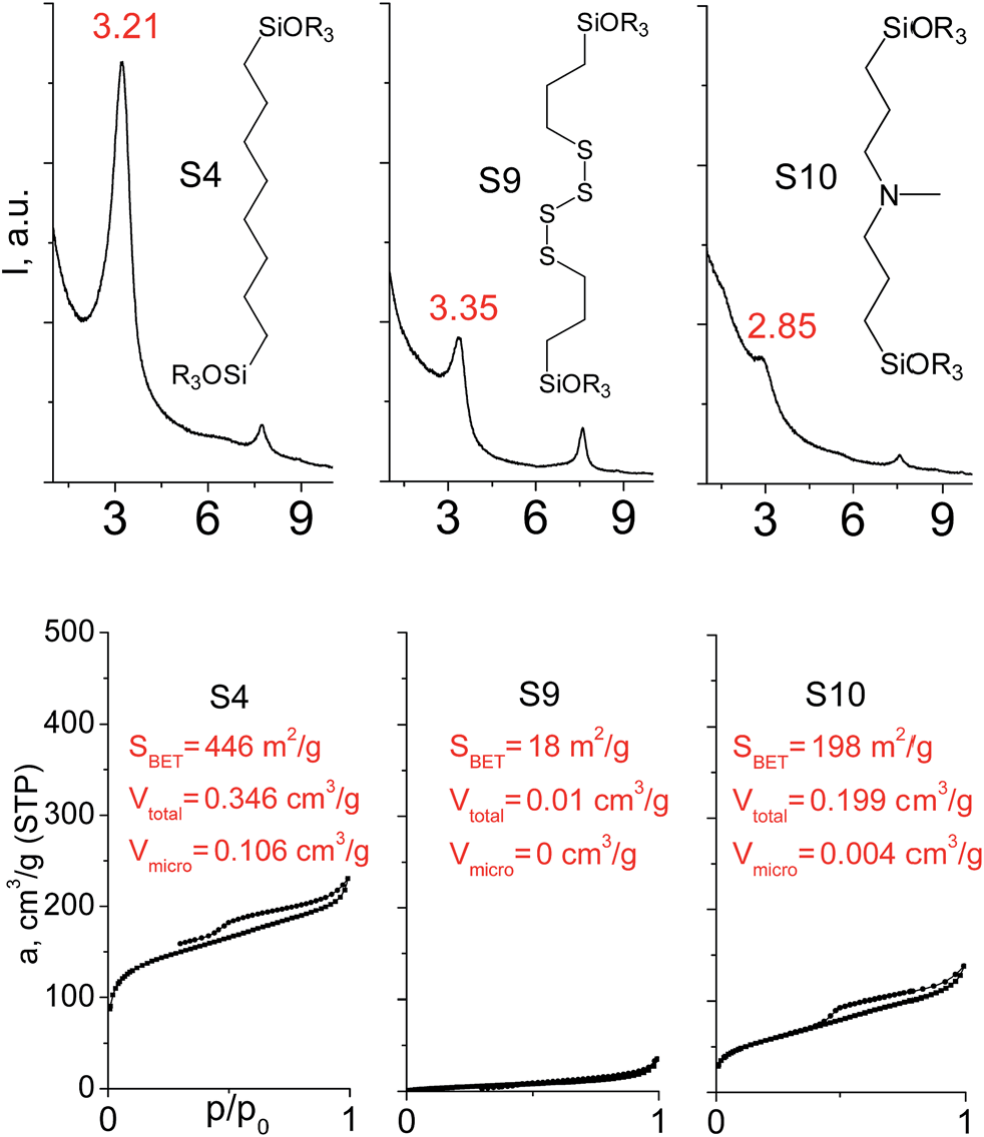
XRD patterns (top), isotherms of nitrogen adsorption and characteristics of porosity (bottom) of hybrid materials obtained using precursors with the similar size, but different in the nature of the organic chain.

In our previous report,^14^ the synthesis of the parent zeolite, its hydrolysis and swelling of obtained layers was discussed in detail. In addition, we confirmed the successful condensation of different types of silsesquioxane molecules with silica layers of IPC-1P and complete removal of swelling agent (CTMA) from the interlayer space using solid state ^31^Si and ^13^C NMR spectroscopy.^14^ Here, we would like to focus on the structural ordering of the formed hybrid materials reflected, for example, by changes in positions and relative intensities of dominant reflections in XRD patterns, as well as the dependence of sorption characteristics on linker type. For that purpose we used organic linkers S4, S9, S10 (Table 1) similar in denticity and size (chains consisted of 7–10 atoms), but different in the nature of their backbone (hydrocarbon, polysulfide-containing and amine). The diffraction patterns of all prepared hybrids contain well-resolved peaks centred around 3°, but their intensities significantly drop when C-atoms in S4 substrate were partially substituted by polysulfide- (S9) and especially amino-groups (S10, Fig. 2). The advantage of hydrocarbon chain became especially pronounced when comparing sorption properties of hybrid materials (Fig. 2). The difference in the behaviour of the linkers under investigation may be connected to the difference in their hydrophilicity determining the distribution of the organic precursor during hydrolysis step between water solution and interlayer space filled with hydrophobic alkyl chains of CTMA. Due to the presence of electron lone pairs in amine and polysulfide chains, respective N- and S-containing precursors can be partially deintercalated from the interlayer space, that leads to the decreased stability of the organic pillars and consequently to their partial (in the case of S10 ligand) or almost complete (S9) collapse after removal of swelling agent (Fig. 2).

**Fig. 3:**
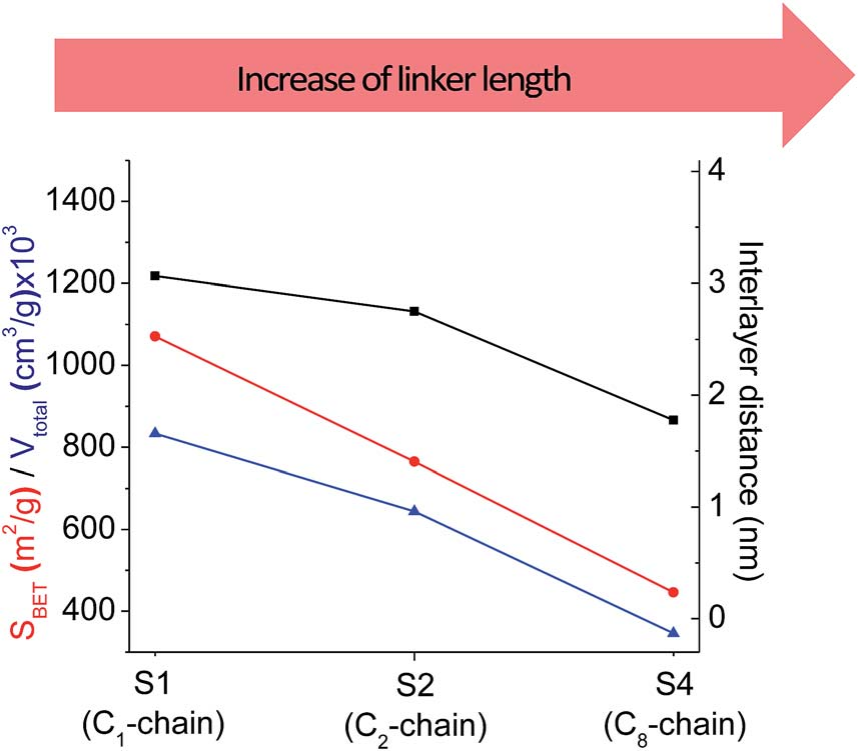
The change of textural and structural parameters of hybrid materials obtained using precursors containing non-branched saturated aliphatic chains different in size.

The increase in the number of carbon atoms in the aliphatic hydrocarbon chain results in monotonic decrease in the interlamellar distance, surface area, and void volume of hybrid materials (Fig. 3). It should be pointed out that since the intercalation step was performed using already expanded (swollen) material, intercalation agent fills the available interlayer space instead of forming layer-agent-layer clusters with only one linker molecule between the layers. Thus, the interlayer distance in materials before SDA removal was comparable (about 2.7–3.0 nm), while final hybrids exhibited significantly different layer expansion (from 1.8 nm for C_8_-chain to 3.1 nm for C_1_-precursor). The changes can be explained by increased flexibility of long carbon chains that allows twisting and shrinkage of the organic pillars resulting in the observed changes in structural (*d*-spacing) and porous (*S*_BET_, *V*_total_) properties.

**Fig. 4:**
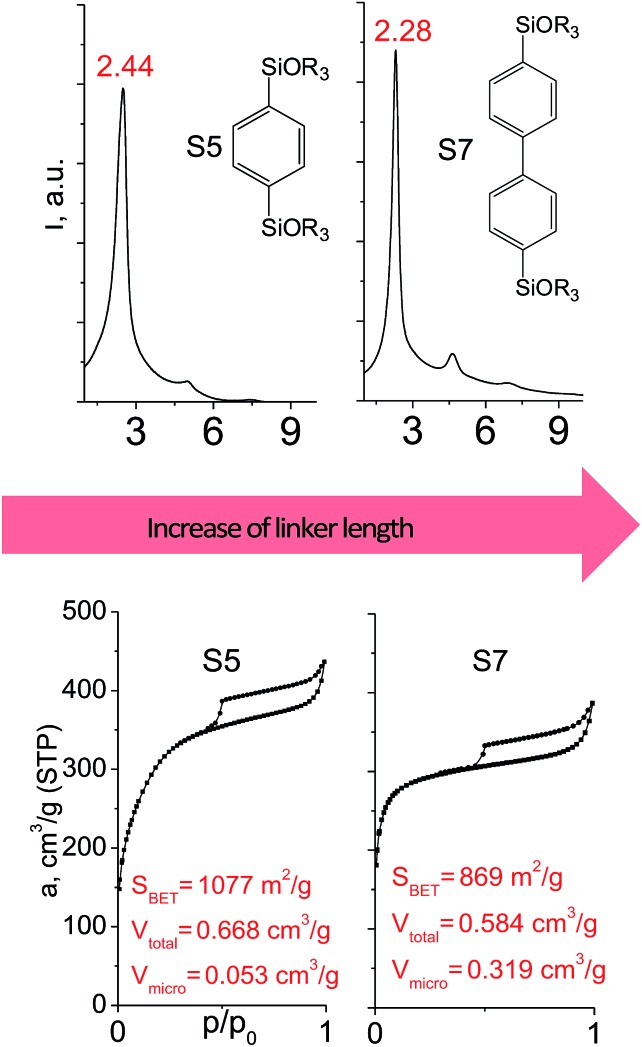
XRD patterns (top), isotherms of nitrogen adsorption and characteristics of porosity (bottom) of hybrid materials obtained using aryl-containing precursors different in size.

In contrast to alkyl-containing silsesquioxanes, the change of the size in aryl-containing linkers (S5, S7, Table 1) did not lead to the dramatic changes in structural and textural properties of respective hybrids (Fig. 4). In particular, position of interlayer diffraction line, shape of the isotherm, surface area and total pore volume did not significantly varied when *p*-phenylene siloxane (S5) was replaced by biphenylene (S7). The reason of the different behavior of aryl-containing linkers in comparison with alkyl analogues probably consists in much higher rigidity of the first ones (will be discussed in more details in Section 3.4). Remarkable growth of micropore volume with increasing aryl chain length (Fig. 4) may indicate the appearance of additional void volume between large linkers.

### 3.2. Denticity of the linker

Denticity of the precursor molecule obviously determines the maximum number of the possible linkages between organic building-block and (i) another siloxane molecules in the pillar or (ii) silicon atoms on the layer surface. The obvious expectation is the deterioration of the stability and porosity of the hybrid materials with the decrease in the denticity of the linkers with the same nature. But the question is, is there a minimum number of alkoxy groups in the linker, which will allow the successful pillaring with the maintenance of reasonable porosity and structural ordering?

**Fig. 5:**
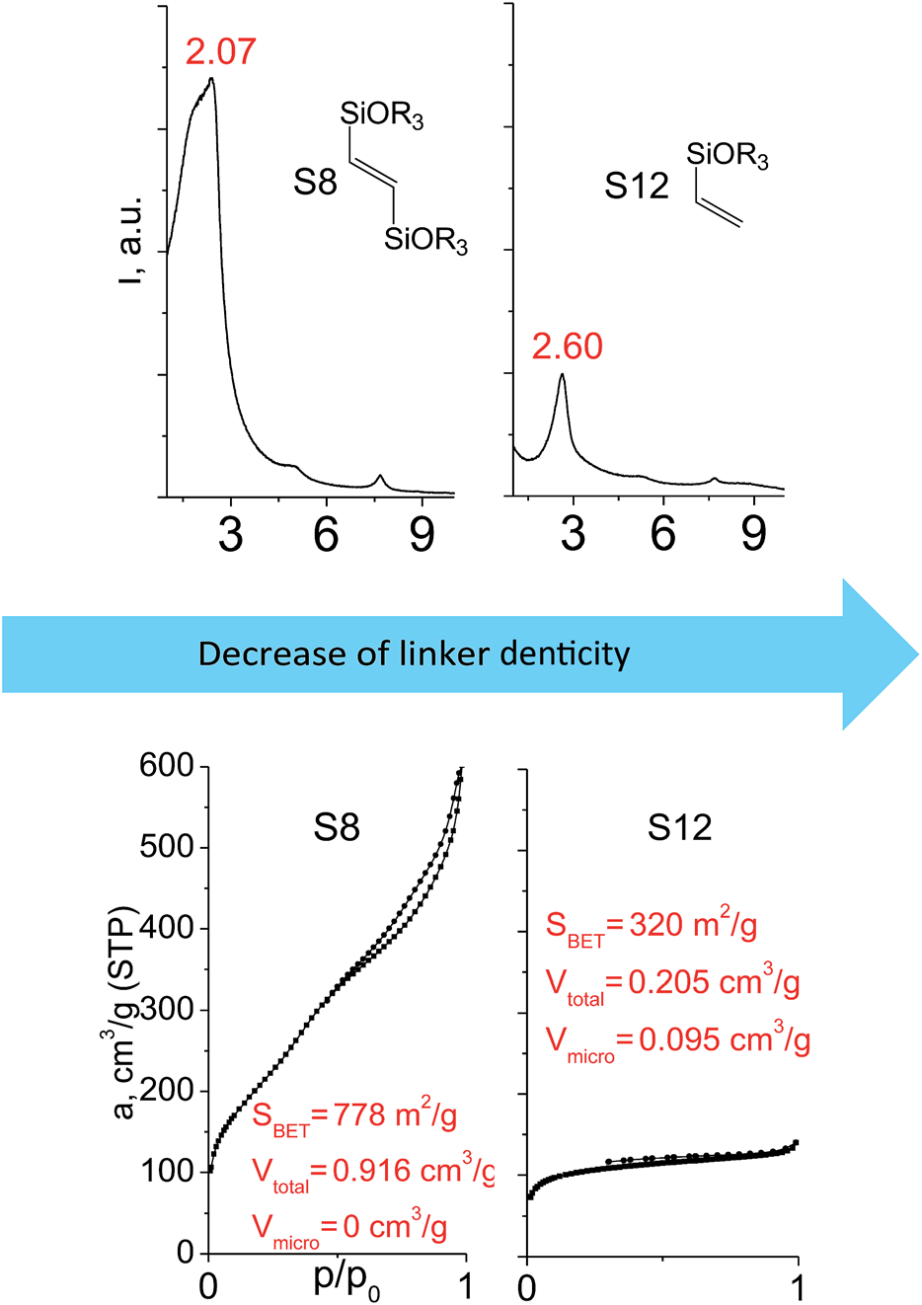
XRD patterns (top), isotherms of nitrogen adsorption and characteristics of porosity (bottom) of hybrid materials obtained using small-size hexadentate bis- (left) and tridentate mono- (right) trialkoxysilyl precursors.

When small-size vinyl compounds were used as linkers (Fig. 5), decrease in the denticity did not lead to a significant disordering of the layer arrangement, but interlayer distance and especially porosity parameters dropped dramatically. The interlayer diffraction line shifted from 2.07 to 2.60°, which corresponds to the layer shrinkage by 0.87 nm. Appearance of significant amount of micropores instead of mesopores (in comparison to S8) as well as the non-matching of adsorption and desorption branches (at *P*/*P*_0_ ≈ 0.3) in the case of S12-containing sample (Fig. 5) indicates high flexibility of the pillars constructed from linker S12, which is characterized by a low-denticity.

**Fig. 6:**
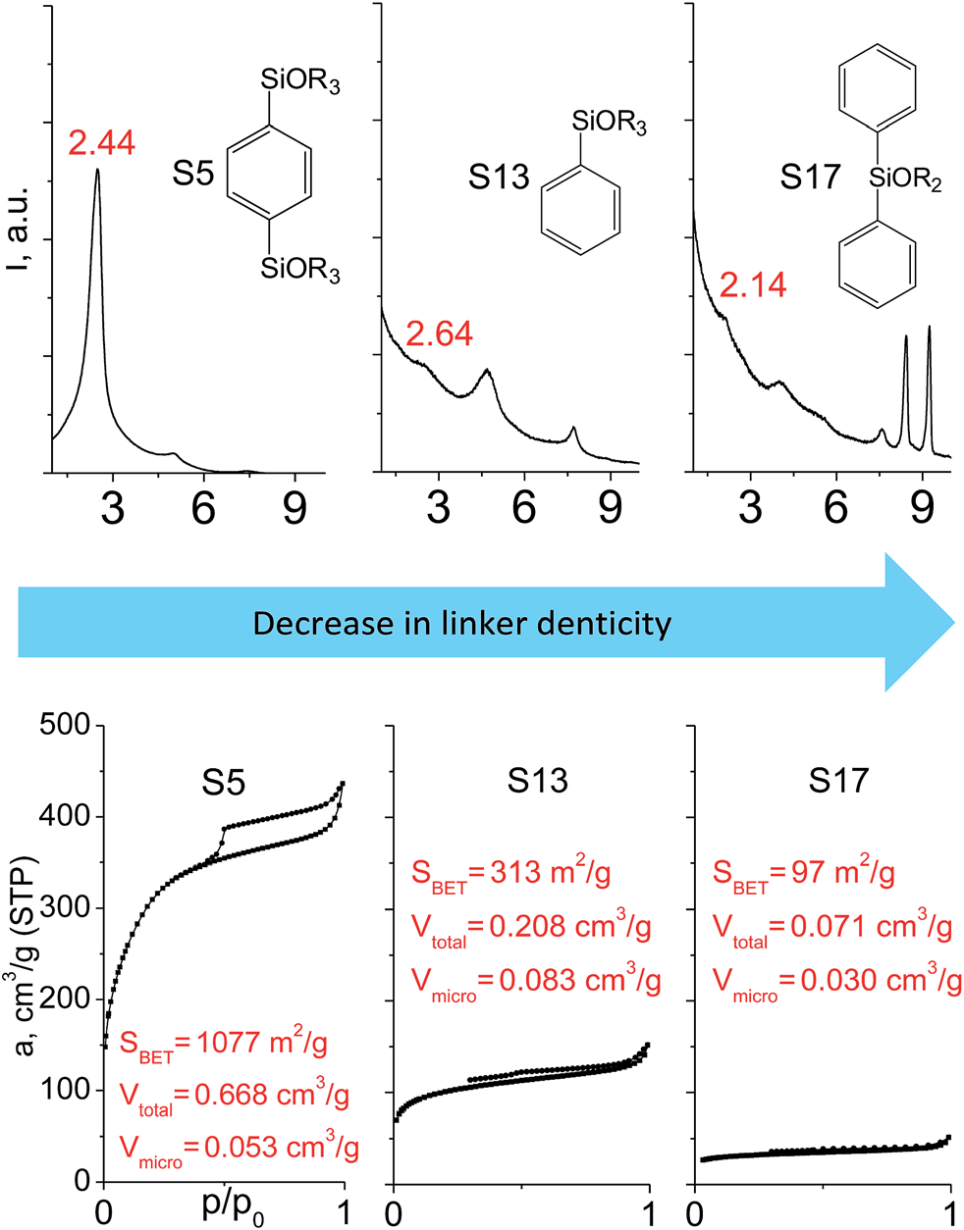
XRD patterns (top), isotherms of nitrogen adsorption and characteristics of porosity (bottom) of hybrid materials obtained using medium-size hexadentate (left), tridentate (middle) and bidentate (right) polyalkoxysilane precursors.

The effect of denticity became even more pronounced with larger aromatic linkers. XRD patterns of the materials obtained using monoalkoxysilyl precursors S13 and S17 possessed only very low intensive interlayer diffraction lines indicating the lacking of the regular layer arrangement (Fig. 6). Instead of interlayer lines, additional peaks in the region 7–10° are probably related to the stacking of aromatic rings. Similar to the vinyl-containing materials, the decrease in the linker denticity from 6 (in S5) to 3 (in S13) and further to 2 (in S17) led to the significant decrease in the porosity of respective materials and appearance of mismatching between isotherm branches (Fig. 6). The use of bidentate S17-based linker resulted in the formation of an almost non-porous hybrid.

**Fig. 7:**
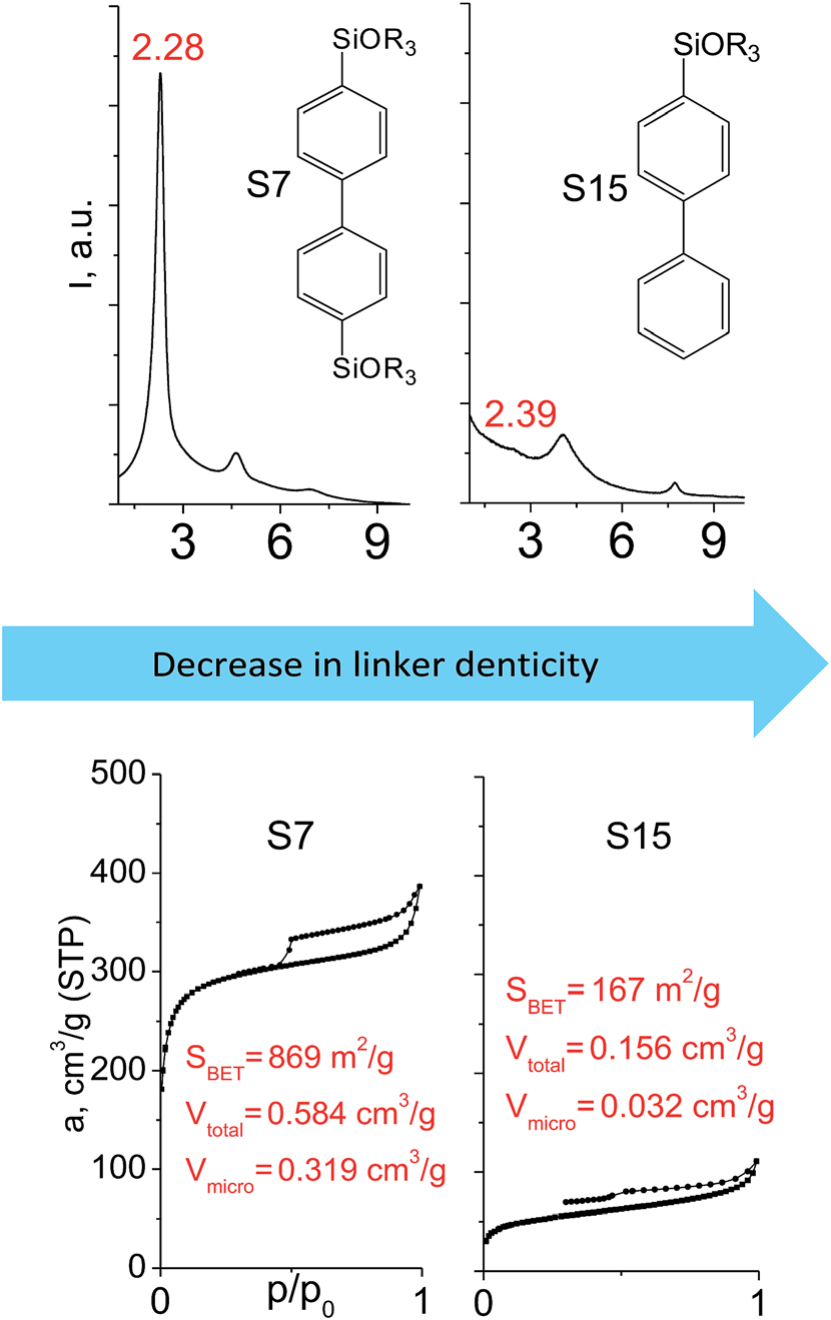
XRD patterns (top), isotherms of nitrogen adsorption and characteristics of porosity (bottom) of hybrid materials obtained using large-size hexadentate (left) and tridentate (right) organic precursors.

The major effect of the alkoxysilyl group number in the organic precursor on the structural and textural parameters of hybrid materials was achieved for the largest aromatic linkers, probably caused by the lowest amount of the intersections formed in pillars. Application of the respective monoalkoxysilyl biphenyl substrate (S15) as a linker resulted in the formation of the hybrid material with the lowest porosity in comparison with dialkoxysilane derivative (S7) having the same organic part (Fig. 7).

The results of this section can be summarized as follows, the effect of linker denticity on the porosity of forming hybrids is dependent on the size of organic precursor. Decrease in the denticity of linker may be used for generation of additional microporosity when relatively small siloxane molecules (allowing the preservation of the reasonable porosity with a low amount of connections) are used as organic precursors. However, the formation of pillars to efficiently retain the interlayer expansion usually requires hexadentate precursors, especially when using bulky linkers.

### 3.3. Nature and size of side chain

**Fig. 8:**
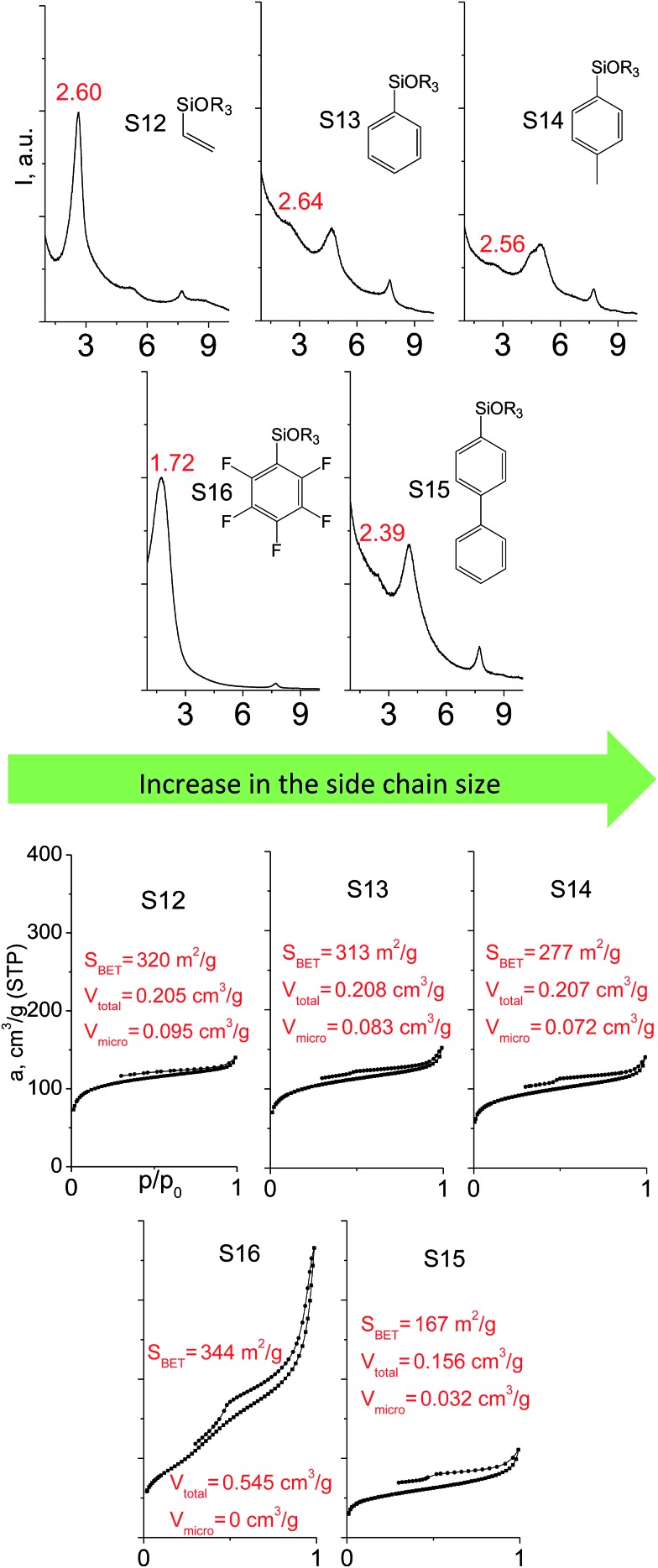
XRD patterns (top), isotherms of nitrogen adsorption and characteristics of porosity (bottom) of hybrid materials obtained using trialkoxysilyl precursors containing substituents different in size.

A set of hybrid materials prepared using unsaturated monoalkoxysilyl linkers was investigated in order to more deeply understand the influence of the linker size. The size of linking molecules increases in the range vinyl- (S12) < phenyl- (S13) < tolyl- (S14) < pentafluorophenyl- (S16) < biphenyl- (S15). Excepting the perfluorinated S16 precursor, linkers under investigation are hydrocarbons not possessing any functional groups that can bring additional interaction between the organic parts of the pillars. Therefore, the sorption characteristics of respective hybrid materials quite predictably decrease with the growth of the substituent bulkiness in the following sequence (Fig. 8): for *V*_total_ as S12 ≈ S13 ≈ S14 > S15 and *V*_micro_ – S12 > S13 > S14 > S15.

Surprisingly, the use of S16 linker characterized by the intermediate size resulted in the formation of an exclusively mesoporous, well-ordered (in comparison to other monoalkoxysilyl analogues) material that exhibited the largest surface area and total pore volume (Fig. 8).

Explanation of this phenomenon may rely on the particularly high hydrophobicity of perfluorinated substituent causing reasonably strong linker–linker interactions that determine optimal self-arrangement of the substrate molecules in the interlamellar space prior to the hydrolysis step. As a result, the final hybrid molecular sieve containing more rigid pillars constructed from S16 not only maintains the high porosity, but also exhibited much larger interlayer distances if compared with other materials mentioned above (Fig. 8).

### 3.4. Rigidity of the chain

**Scheme 1:**
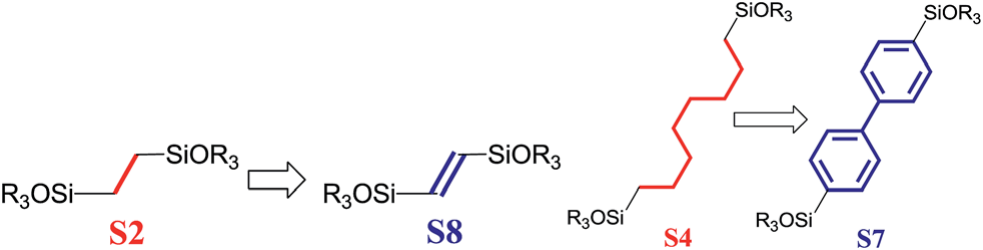
A comparison of ligands of different rigidity used in this study.

To investigate the direct influence of the linker rigidity on the physico-chemical properties of respective organic–inorganic materials, the short-chain (C_2_) and long-chain (C_8_) precursors have been chosen (Scheme 1). We compared saturated alkyl-containing precursor (S2) with vinyl analogue (S8), and a flexible linker (S4) with an aromatic one (S7).

In contrast to S2 and S4, both S7 and S8 linkers have no rotational degrees of freedom affecting the geometry of the chain (biphenylene chain may be considered as a cylinder due to the high rate of the aromatic ring rotation because of a low potential barrier for this process at room temperature^24^). Thus, organic chains consisted of exclusively sp^2^ carbon atoms (in S7 and S8) represent the completely rigid analogues to respective aliphatic compounds.

**Fig. 9:**
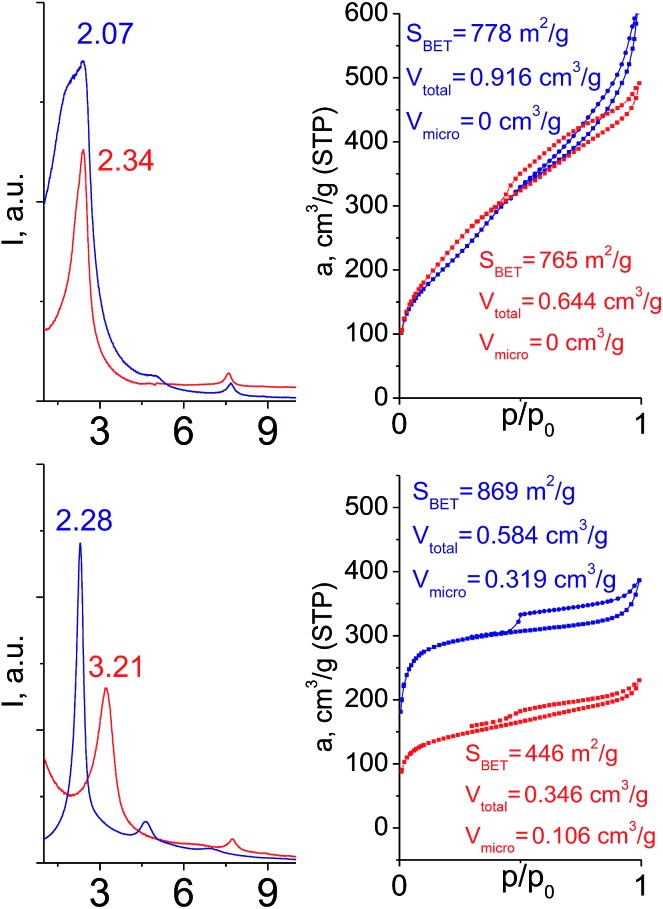
Influence of the linker rigidity on the position of interlayer (200) reflection and adsorption capacity of hybrid materials.

Hybrids prepared with linkers having relatively short organic chains (C_2_ in S2–S8 pair) show similar structural (position and relative intensity of the interlayer reflection) and sorption properties (shape of the isotherm and *S*_BET_/*V*_total_ characteristics) (Fig. 9). The noticeable difference consists in the enhanced volume of large mesopores and macropores (0.8 > *P*/*P*_0_ > 0.95) when more rigid vinylene linker have been used.

In contrast, a significant decrease in the interlayer distance and sorption capacity together with a mismatch between adsorption and desorption branches of the isotherm (indicating the low rigidity of the pillars, Fig. 9) was found for a hybrid bearing a more flexible aliphatic chain (C_8_ in S4) compared with one possessing a more rigid S4 linker. However, the shape of the isotherm and quite high relative content of the micropores were found to be comparable for both materials.

### 3.5. Nature and size of condensing group

Different kinds of alkyl substituents in alkoxysilyl groups can act as leaving groups during the hydrolysis step (5 on Fig. 1). Although the chemical nature of such groups is almost the same (for example, if methyl group is replaced by ethyl), the size of the respective condensing group increases significantly. It can lead to decreased concentration of organic precursor in the interlamellar space at intercalation step (4 on Fig. 1) and therefore it may reduce the stability of the subsequently formed pillars. To investigate this feature, we compared the properties of hybrids obtained using triethoxysilyl- (S2) and trimethoxysilyl-containing (S3) organic precursors under the same conditions.

**Fig. 10:**
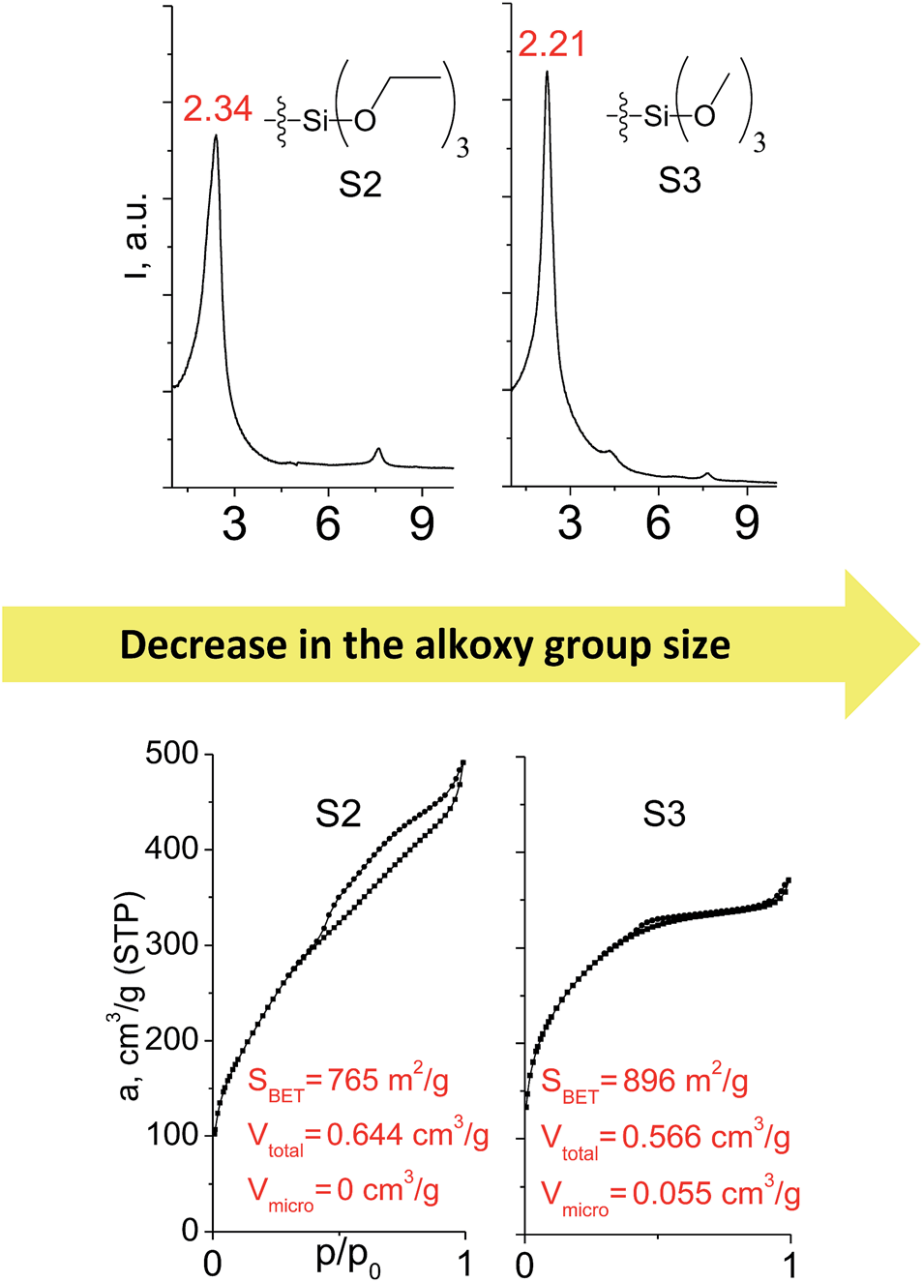
XRD patterns (top), isotherms of nitrogen adsorption and characteristics of porosity (bottom) of hybrid materials obtained using organic precursors containing ethyl (left) and methyl (right) leaving groups.

Fig. 10 shows similar distances (2.34 *vs.* 2.21 Å) between inorganic layers in both materials while apparent differences in sorption behavior can be noticed. In particular, the use of S2 as a precursor resulted in the formation of the hybrid with very wide pore size distribution in the adsorbate pressure range up to 0.95 *P*/*P*_0_ (PSD plot is not presented due to the absence of well-resolved peaks on d*V*/d*r*–*r* curve) causing appearance of a H3-type hysteresis loop characteristic for highly heterogeneous pore networks with large mesopores and non-rigid walls.^25^ At the same time, the sorption isotherm for the S3 material possesses only a negligible hysteresis loop, while the main part of adsorbate uptake proceeds at lower relative pressure *P*/*P*_0_ of 0.4. It indicates the presence of relatively small and narrow mesopores (*D*_pore_ < 4 nm) as a dominant channels in the pore system of S3-hybrid as well as some amount of micropores. The different behavior of the precursors possessing diverse leaving groups may be explained by more dense filling of interlayer space with S3 precursor, which leads to the increasing amount of intrapillar intersections and therefore to the maintenance of medium-size pores after SDA removal. On the other hand, S2 provides higher flexibility of the pillars that may lead to their partial collapse and increasing diameter of remaining mesopores.

**Fig. 11:**
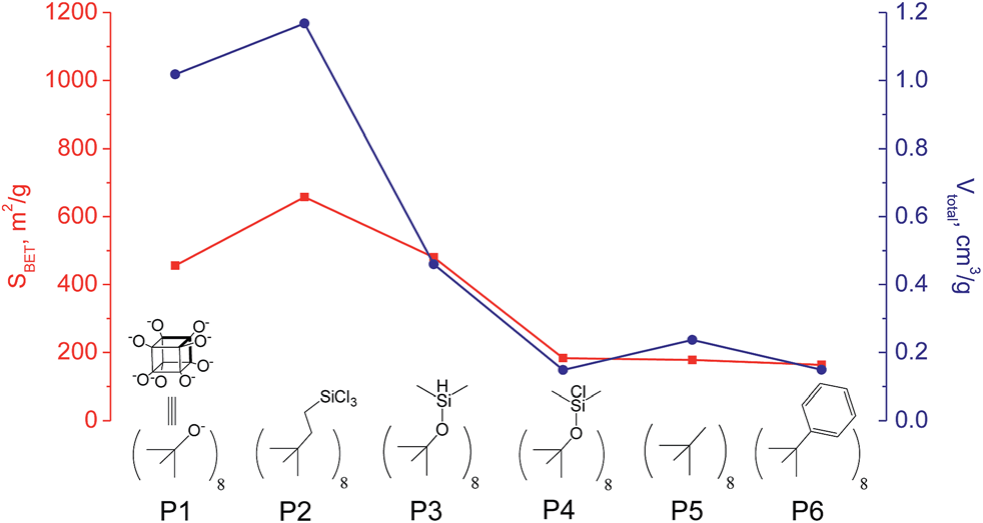
Change of surface area and total pore volume of hybrid materials obtained using different types of polyhedral oligomeric siloxanes.

The use of polyhedral oligomeric silsesquioxanes and silicates (P1–P6, Table 1) possessing different types of leaving groups allowed us to obtain hybrid materials with diverse porosity. However, most of them were characterized by the absence of structural ordering (Fig. S1†). In particular, substrates with OH/H leaving group (P1, P3) or large amount of Cl leaving groups (P2) gave much higher *S*_BET_ and *V*_total_ (Fig. 11). Since the Si–Cl bond is more stable than Si–H (the difference is equal to 65 kJ mol^–1^ (ref. 26)), the construction of the P4-based pillars may be complicated in comparison with P3 ones, that probably is the reason of much lower porosity of hybrid synthesized using P4 precursor. Although Si–C bonds in P5 and P6 linkers can be hydrolysed under applied conditions^27^ allowing their condensation with inorganic layer, a low rate of this process and absence of leaving groups (*e.g.* OR, Cl *etc.*) may limit the efficient pillaring and cause very low porosity of respective materials (Fig. 11).

### 3.6. Organic precursor/inorganic layers ratio

Stability of the pillars fabricated in the interlamellar space of IPC-1P is expected to depend on (i) a thickness of organosilica domains, (ii) their packing density (local concentration), and (iii) number of intersections (dependent on the size and denticity of the precursor). Parameters (i) and (ii) can easily be varied by changing the ratio between linker and inorganic sheets.

In this section the effect of w/w precursor/layers ratios (*i.e.*, 1 : 0.5; 1 : 1; 1 : 1.5) on the structural and textural properties of UTL-derived hybrids will be discussed.

**Fig. 12:**
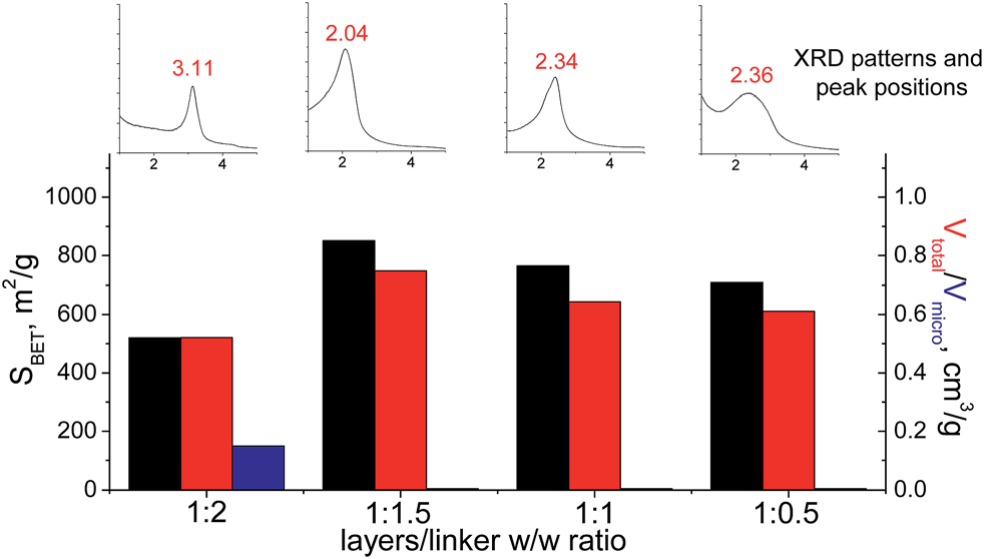
Change of structural and textural properties of hybrid materials obtained using different ratios [layered silica precursor]/[S2 organic precursor].

In the case of small-size linker (S2), the optimal linker/layer ratio was 1 : 1.5, since the respective hybrid material possesses the highest interlayer distance (3.3 nm), surface area (850 m^2^ g^–1^), and mesopore volume (0.75 cm^3^ g^–1^). The lower amount of linker appeared to be not so efficient for construction of the pillars rigid enough for maintenance of high porosity (Fig. 12). At the same time, the further growth in the content of the organic precursor resulted in substantial decrease in the total pore volume and appearance of micropores due to the significant decrease in the interlayer distance (to 1.8 nm). Such shrinkage of the interlayer distance may be connected with a partial deintercalation of the swelling agent (CTMA) caused by its replacement with the organic precursor at the impregnation step (4 on Fig. 1).

The similar trend was observed when aromatic linkers (S5 and S7, Table 1) have been applied (Fig. S2 and S3†). In these cases, 1 : 1 organic/inorganic components ratio was the optimal one, which is most probably related to much higher rigidity of the aromatic chains allowing to use a lower amount of building-blocks for efficient pillaring in comparison to aliphatic substrate (S2).

**Fig. 13:**
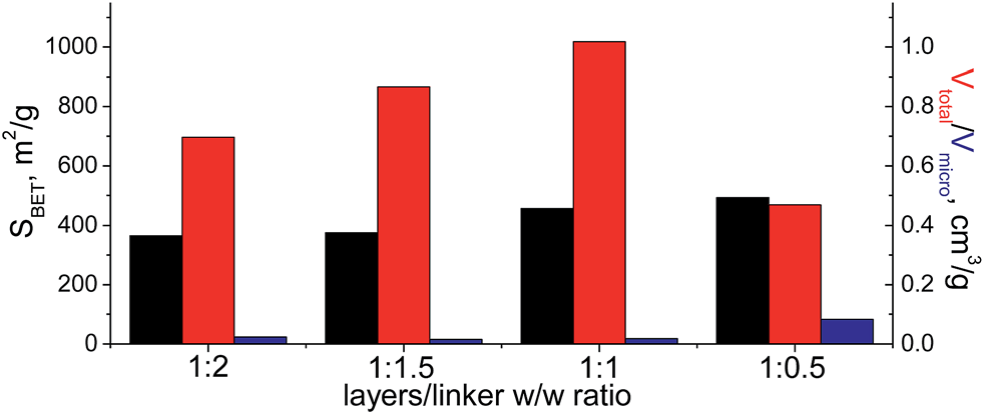
Change of structural and textural properties of hybrid materials obtained using different ratios [layered silica precursor]/[P1 precursor].

When bulky P1 precursor was used (Fig. 13), we noticed only small change of the surface area (in the range 370–495 m^2^ g^–1^) with variation of oligomeric silane content, while void volume for investigated samples differ more significantly (in the range 0.470–1.015 cm^3^ g^–1^). As in the previous case, the optimal content of the linker has been achieved at 1 : 1 ratio.

### 3.7. Addition of “binder”

In particular cases we used a mixture of respective organic linker and “binder” to prop the interlayer space of layered precursor. The idea consists of the use of relatively low-molecular additive with high denticity (TEOS) to increase the stability of hybrid pillars when materials possess poor ordering/porosity caused by features of the respective linker (long chain, low denticity, bulkiness).

**Fig. 14:**
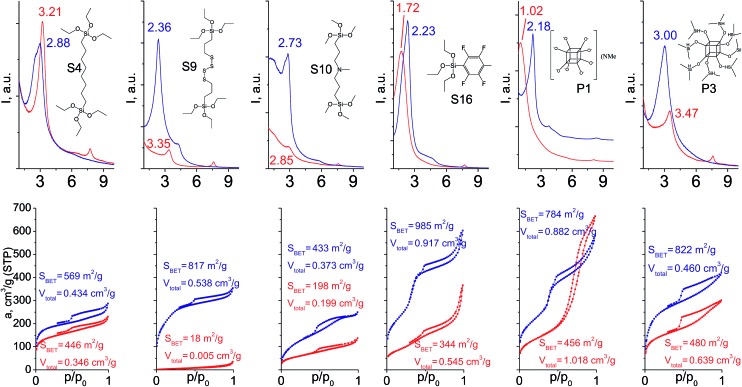
XRD patterns (top) and N_2_ sorption isotherms (down) of hybrid materials obtained without (red) and with addition of TEOS (blue).

When addition of TEOS was used (Fig. 14), the relative intensity and width of the interlayer reflections either remained quite similar in comparison to “binder”-free hybrids (S4, S16) or improved significantly (S9, S10, P1, P3). Noticeably, the positions of all these reflections became much closer to the value 2*θ* = 2.5–2.6° characteristic for swollen material^28^ used for intercalation of organic precursors. It indicates an enhanced stability of “binder”-assisted pillars against shrinkage or expansion after CTMA removal.

In the case of materials that originally possessed reasonable porosity (materials derived from S4, S16, P1, P3, Fig. 14), the addition of TEOS did not improve textural properties of the respective hybrids as dramatically as in the case of bulky heteroelement-containing substrates (*e.g.* the growth of *S*_BET_ for S4 hybrid from 446 to 569 m^2^ g^–1^ was observed, when for S9 analogue it increased from 18 to 817 m^2^ g^–1^). For S9 and S10 linkers, the use of “binder” was crucial for the fabrication of porous materials (Fig. 14). Except for the hydrophilic P1 precursor, hybrids synthesized both with/without addition of TEOS were characterized by the same type of sorption isotherm, that indicates the same way of linker–CTMA and linker–linker interactions on the intercalation step resulting in the similar distribution of the hydrophobic precursor (S*x*, P*x*, TEOS) in the interlayer space.

The combination of such factors as high denticity of TEOS and hydrophobic interactions of pentafluorophenyl rings with each other provided uniformly ordered materials having apparent step on the adsorption isotherm (S16 on Fig. 14) indicating narrow pore size distribution (≈2.5 nm). Textural properties of this material significantly exceed those for its analogues (for example, *S*_BET_ was equal to 985 m^2^ g^–1^ for S16-based hybrid, while surface area of materials obtained using other tridentate precursors did not exceed 350 m^2^ g^–1^).

Addition of TEOS for facilitation of the P1 precursor condensation resulted in a dramatic increase in the structure ordering and right-shift of interlayer diffraction line (Fig. 14). Such significant change of *d*-spacing (from 8.6 nm to 4.0 nm for materials synthesized without and with “binder”, respectively) may be explained by the change of the mechanism of P1 precursor intercalation in these two cases. In particular, when pure oligomeric silicate salt P1 is used, high polarity of its molecules assumedly prevent the efficient interaction of the linker and hydrophobic chains of CTMA located between the inorganic layers, that may result in limited interpenetration of the surfactant and precursor phases. After pre-condensation of TEOS and P1 due to the increased hydrophobicity of the oligomer species, their distribution in the interlayer space is significantly improved.

Therefore, hybrid prepared in the presence of TEOS was characterized by uniform pores with diameter 3–3.5 nm usual for samples pillared with silsesquioxanes in contrast to the material obtained using exclusively P1 precursor for pillaring (characterized by pore size distribution in the range 5–10 nm).

### 3.8. Microstructure of pillared hybrid materials

**Fig. 15:**
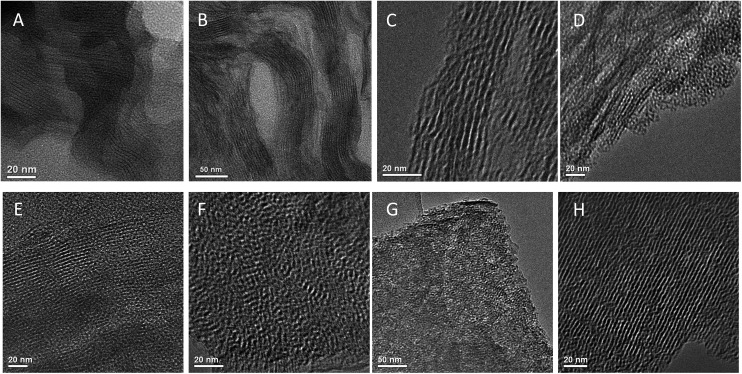
HRTEM images of swollen IPC-1P layers (A) and hybrid materials obtained using S2 (B), S4 (C), S16 (D – without TEOS, E – with TEOS), P3 (with TEOS, F) and P1 (G – without TEOS, H – with TEOS) precursors.

To follow the changes in arrangement of the layers when soft alkyl- or more rigid aryl-containing linker were used, as well as changes caused by using of additional “binder” (*i.e.* TEOS), HRTEM images of respective samples were collected (Fig. 15). Noticeably, the initial inorganic layers were not completely rigid in the swollen form (Fig. 15A) that was also observed for alkyl S2-based material (Fig. 15B). However, respective hybrid still possesses reasonably large domains of straight inorganic sheets similarly to aryl-containing sample (S4, Fig. 15C).

In agreement with the results of XRD, hybrid materials synthesized without using of “binder” (*e.g.* TEOS) were characterized by significantly lower layer ordering and presence of a large amount of amorphous phase (Fig. 15D and G) in comparison with their analogues obtained by addition of TEOS (Fig. 15E–H). In latter case, HRTEM images evidence the quite uniform interlayer distances in synthesized material. Most of layers packed by several tens units and they seems to be more rigid than in hybrids mentioned above. As it follows from HRTEM images, average basal spacing in “binder”-assisted materials under investigation varied in the range 3–4 nm.

### 3.9. Application of hybrid materials

Since obtained hybrid organic–inorganic materials possess different porosity (size of the channels, pore volume) and functionality determined by the nature of the linker, they can be used for various applications related to sorption and catalysis. To confirm the possibility for tuning of the catalytic properties of obtained hybrids, several representative samples (S4 and S10 obtained with addition of TEOS) have been compared with purely siliceous, pillared material IPC-1PI (synthesized similarly to hybrid samples but using only TEOS instead of silsesquioxanes) in a condensation reaction between benzaldehyde and malononitrile. All chosen catalysts characterized by comparable pore volume (0.434 cm^3^ g^–1^ for S4, 0.373 cm^3^ g^–1^ for S10 and 0.541 cm^3^ g^–1^ for IPC-1PI) and surface area (569 m^2^ g^–1^ for S4, 433 m^2^ g^–1^ for S10 and 701 m^2^ g^–1^ for IPC-1PI), but different chemical nature of the connectivity between zeolite layers (purely aliphatic chains in S4, N-containing S10 and inorganic pillars in IPC-1PI). This difference is crucial for catalytic activity: while material not possessing functional groups show very low conversion of benzaldehyde (<5% after 2 h), S10-based material exhibited almost 100% yield of target benzylidenemalononitrile under the same reaction conditions. This result is an example of general possibility for adjustment of chemical and textural properties of micro-mesoporous organic–inorganic materials, which can be used for practical application.

## 4. Conclusions

Porous organic–inorganic UTL-derived materials with tunable textural characteristics were synthesized using silsesquioxanes and polyhedral oligomeric siloxanes differing in nature. The selection criteria of organic molecules appropriate for use as a linker allowing the preparation of hybrids possessing high sorption characteristics can be summarized as follows:

• the use of silsesquioxanes possessing hydrophobic alkyl chain favors the formation of ordered micro-mesoporous hybrids, while partial deintercalation of more hydrophilic polysulfide and amine-containing linkers on the hydrolysis step result in formation of materials with lower porosity.

• The increase in carbon atoms number in the aliphatic hydrocarbon chain of silsesquioxane linkers leads to monotonic decrease of the interlamellar distance, surface area and void volume of hybrid materials, connected with shrinkage of forming organic pillars. In contrast to alkyl-containing silsesquioxanes, the increase of the chain size of aryl-containing linkers leads to remarkable growth of micropore volume due to the high rigidity of aryl chains.

• The effect of linker denticity on the porosity of forming hybrids is dependent on the size of organic precursor. Decrease in denticity of linker may be used for generation of additional microporosity when relatively small siloxane molecules are used as organic precursors. Although, the formation of the pillars enable efficiently retain interlayer expansion usually require hexadentate precursors, especially when using bulky linkers.

• While not influencing textural characteristics of hybrids prepared using short-chain silsesquioxanes (*e.g.* C_2_), the increase of flexibility of aliphatic chain in comparison to aromatic analogue provoke significant decrease of the interlayer distance and sorption capacity when using long-chain (*e.g.* C_8_) silsesquioxanes.

• Textural characteristics of prepared hybrids increases with decreasing the size of leaving alkoxy-group (*e.g.* EtO– > MeO–), while decreasing in following sequence of leaving groups in polyhedral oligomeric silanes: –OH > –H > –Cl > –Me ≈ –Ph.

• The use of mixtures of silsesquioxanes and TEOS resulted in increasing specific pore volume and surface area of hybrid samples, caused by increasing number of intermolecular bonds within pillars.

## Supplementary Material

Supplementary information
