# Supplementary material for: Zeolite-derived hybrid materials with adjustable organic pillars
Source: Chem Sci. 2016 Feb 9;7(6):3589–601. doi: 10.1039/c5sc04602e (PMC6008708; doi:10.1039/c5sc04602e)
Supplement: Supplementary file 1 [file SC-007-C5SC04602E-s001.pdf]

Electronic Supplementary Material (ESI) for Chemical Science.
This journal is © The Royal Society of Chemistry 2016

Supporting Information

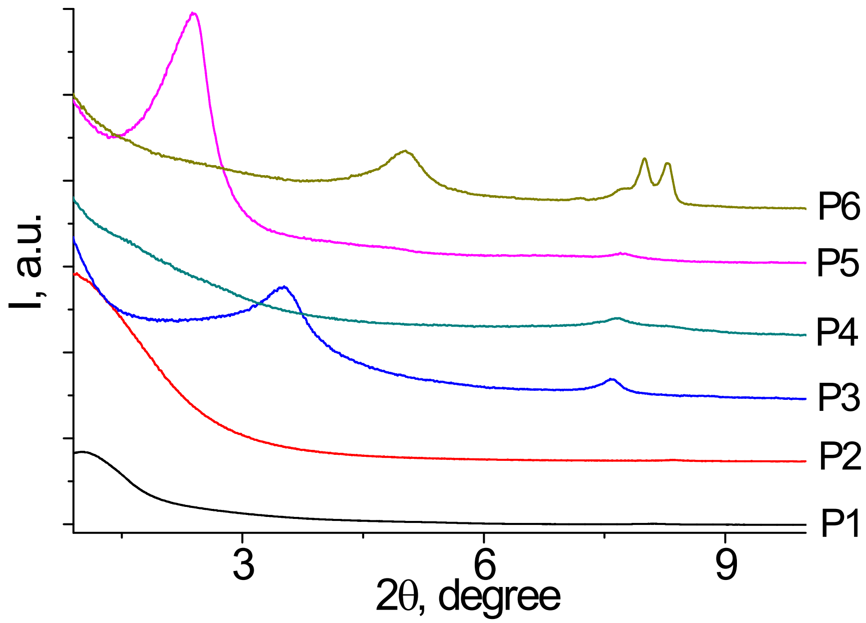

Fig. SI-1. XRD patterns of hybrid materials obtained using different types of polyhedral oligomeric siloxanes.

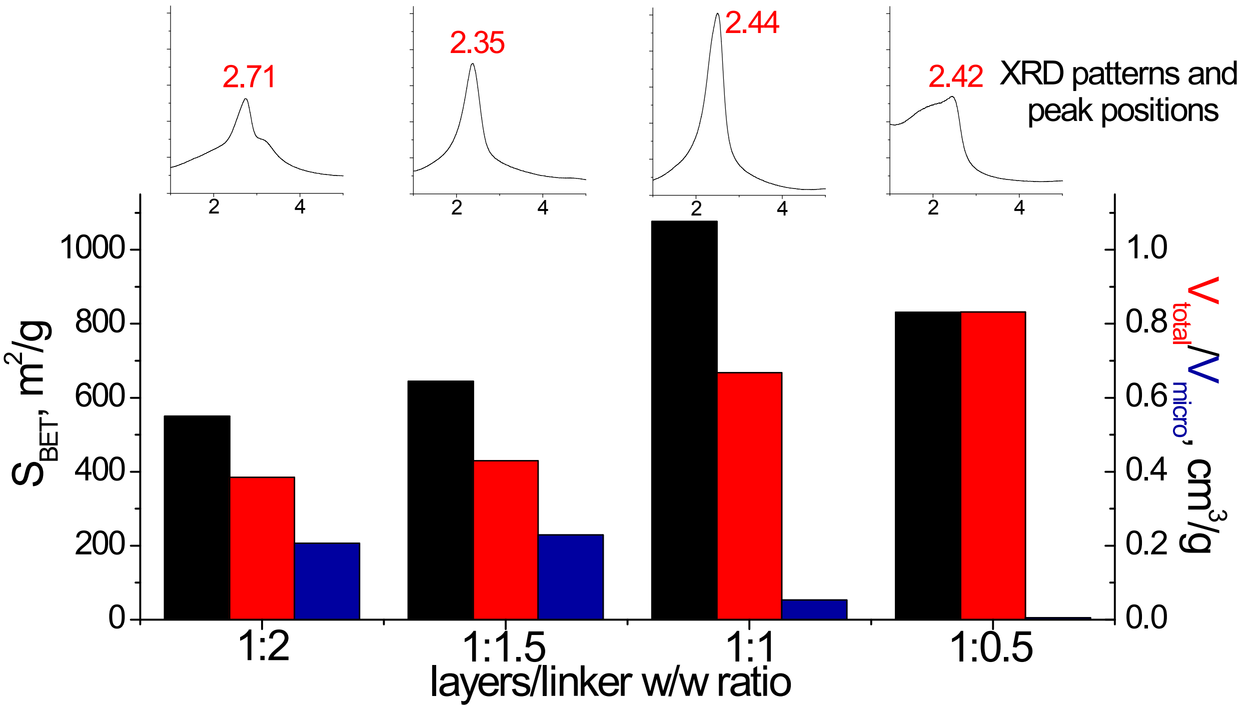

Fig. SI-2. Change of structural and textural properties of hybrid materials obtained using different ratios [layered silica precursor]/[S5 organic precursor].

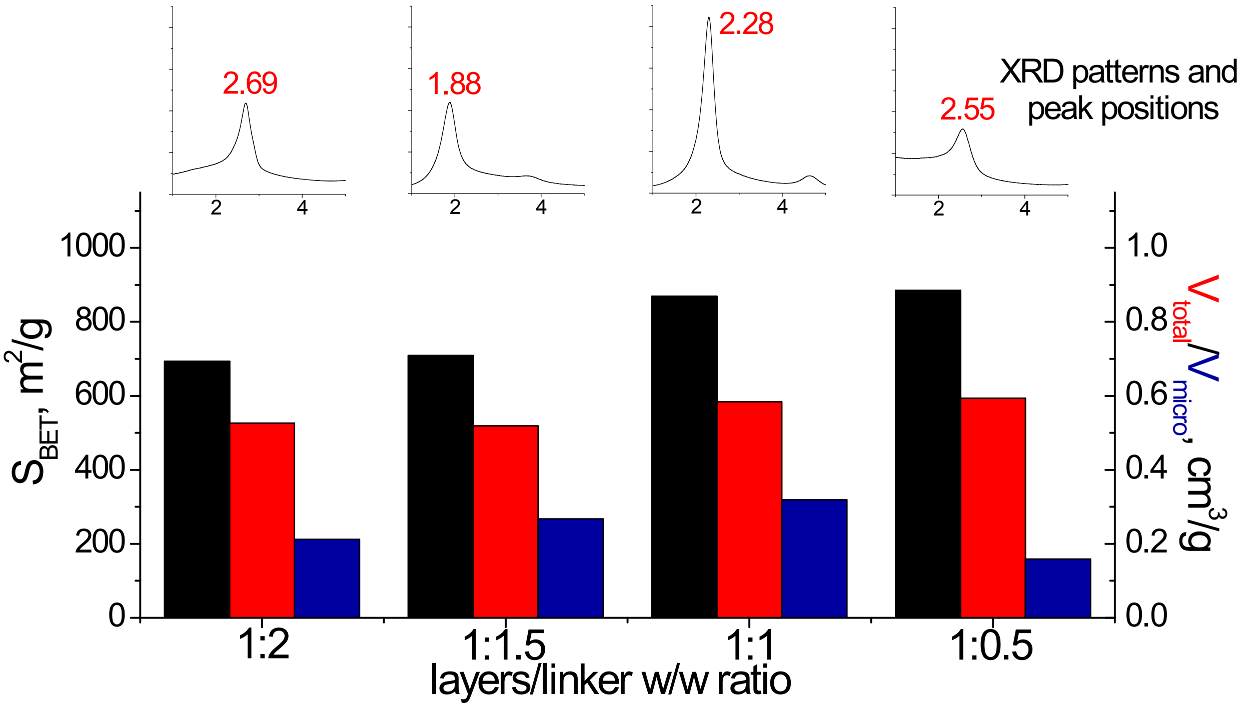

Fig. SI-3. Change of structural and textural properties of hybrid materials obtained using different ratios [layered silica precursor]/[S7 organic precursor].
